# Real-World Comparison of Balloon-Expandable Valves for Transcatheter Aortic Valve Replacement: Data From the Aortic+Mitral TRAansCatheter Valve Registry

**DOI:** 10.1016/j.shj.2026.101104

**Published:** 2026-07-27

**Authors:** Yeela Talmor-Barkan, Kush P. Patel, Marco Barbanti, Sofia Sammartino, Giuliano Costa, Carmelo Grasso, Ole De Backer, Davorka Lulic, Carmen Olmos, Giulia Masiero, Marianna Adamo, Dabit Arzamendi, Mario García-Gómez, Jose A. Baz, Klemen Steblovnik, Victor Mauri, Matti Adam, Hendrik Wienemann, Manuel Hein, Philipp Ruile, Bing Wei Thaddeus Soh, Luca Branca, Rodrigo Estévez-Loureiro, Ignacio J. Amat-Santos, Darren Mylotte, Matjaz Bunc, Giuseppe Tarantini, Michael J. Mullen, Luis Nombela-Franco, Leor Perl, Ran Kornowski, Amos Levi, Guy Witberg

**Affiliations:** a Department of Cardiology, Rabin Medical Center, Petach Tikva, Israel; b Gray Faculty of Medical and Health Sciences, Tel Aviv University, Tel Aviv, Israel; c Barts Heart Centre, St Bartholomew’s Hospital, London, UK; d Cardiology Department, Università degli Studi di Enna Kore, Enna, Italy; e Division of Cardiology, University of Catania, Catania, Italy; f The Heart Center, Rigshospitalet, Copenhagen University Hospital, Copenhagen, Denmark; g Instituto Cardiovascular, Hospital Clínico San Carlos, Instituto de Investigación Sanitaria del Hospital Clínico San Carlos (IdISSC), Madrid, Spain; h Universidad Europea de Madrid, Departamento de Medicina, Facultad de Medicina, Salud y Deportes, Madrid, Spain; i Department of Cardiac, Thoracic and Vascular Sciences, University of Padua Medical School, Padua, Italy; j Cardiovascular Department, Spedali Civili, Brescia, Italy; k Cardiology Department, Hospital de Sant Creu i Sant Pau, Barcelona, Spain; l CIBERCV, Hospital Clínico Universitario de Valladolid, Valladolid, Spain; m Servicio de Cardiología, Hospital Álvaro Cunqueiro, Vigo, Pontevedra, Spain; n Department of Cardiology, University Medical Centre Ljubljana, Ljubljana, Slovenia; o Clinic III for Internal Medicine, Faculty of Medicine and University Hospital Cologne, University of Cologne, Cologne, Germany; p Department of Cardiology and Angiology, University Heart Center Freiburg–Bad Krozingen, Faculty of Medicine, University of Freiburg, Freiburg, Germany; q Department of Cardiology, Galway University Hospitals; and School of Medicine, University of Galway, Galway, Ireland

**Keywords:** Aortic stenosis (AS), Balloon-expandable valves (BEV), Paravalvular leak (PVL), Transcatheter aortic valve implantation (TAVI)

## Abstract

**Background:**

Comparative real-world data on newer balloon-expandable valve (BEV) platforms remain limited. We sought to compare clinical, procedural, and early hemodynamic outcomes following transcatheter aortic valve replacement (TAVR) using 2 contemporary BEVs in a multicenter international registry.

**Methods:**

Consecutive patients undergoing TAVR with Sapien 3/Ultra and MyVal from the Aortic+Mitral TRAansCatheter Valve Registry were included. Propensity score matching was used to generate a balanced 1:3 cohort. The primary endpoint was 2-year all-cause mortality. Secondary endpoints included in-hospital complications, defined according to Valve Academic Research Consortium–3 criteria.

**Results:**

A total of 1903 patients undergoing TAVR with BEV were included (Sapien, n = 1676; MyVal, n = 227). Baseline clinical and echocardiographic characteristics were largely comparable between groups. In the propensity-matched cohort (n = 743), 2-year all-cause mortality did not differ between groups. In-hospital mortality and major procedural complications were similar across platforms. New permanent pacemaker implantation occurred more frequently with MyVal compared with Sapien (26.6% vs. 10.1%; *p* < 0.001). Postprocedural mean transvalvular gradients were comparable between valves, but a greater proportion of Sapien recipients had elevated mean gradients (>10 mmHg), (44.0% vs. 32.3%; *p* = 0.019). Paravalvular leak (PVL) distribution differed, with more-than-mild PVL observed more frequently in the MyVal group (9.8% vs. 2.1%; *p* < 0.001).

**Conclusions:**

In this analysis, both BEV platforms demonstrated comparable safety, mortality, and overall hemodynamic performance following TAVR. However, MyVal was associated with higher rates of permanent pacemaker implantation and PVL. These findings highlight important platform-specific differences that may inform valve selection and warrant further long-term evaluation.

## Highlights

• Real-world Sapien vs. MyVal balloon-expandable transcatheter heart valve (BE-THV) comparison showed similar stroke, vascular, and bleeding rates.
• Two-year all-cause mortality was comparable between both BE-THV platforms.
• MyVal BE-THV was associated with significantly higher permanent pacemaker implantation rates.
• MyVal BE-THV demonstrated higher rates of more-than-mild paravalvular leak postprocedure.
• Sapien BE-THV recipients had higher rates of elevated mean transvalvular gradients (>10 mmHg).

## Introduction

Transcatheter aortic valve (AV) replacement (TAVR) has become the standard of care for patients with severe, symptomatic aortic stenosis across a wide spectrum of surgical risk, owing to its minimally invasive nature and clinical outcomes comparable to surgical AV replacement.^1^^,^^2^ As indications for TAVR expand to younger and lower-risk populations,^3^ device selection, valve durability, and hemodynamic performance have become increasingly important considerations.

Among transcatheter heart valve (THV) platforms, balloon-expandable valves (BEVs) are widely used worldwide. The Sapien valve series (Edwards Lifesciences, Irvine, California) is the most extensively studied BEV, supported by robust randomized and real-world evidence demonstrating favorable procedural safety, clinical outcomes, and valve hemodynamics.^3^ Consequently, Sapien valves are widely regarded as the benchmark BEV platform in contemporary TAVR practice.

More recently, the MyVal BEV (Meril Life Sciences, Vapi, India) has been introduced as an alternative BEV platform.^4^ These devices offer expanded sizing matrices, including intermediate valve sizes and larger diameters, as well as alternative frame and leaflet designs aimed at optimizing annular fit and hemodynamic performance across a broader range of AV anatomies. Although these design features are conceptually attractive, particularly in patients with borderline or large annuli, comparative clinical data against established BEV remain limited.

Although randomized evidence comparing contemporary BEV platforms has begun to emerge (e.g., the COMPARE-TAVI 1 trial),^5^ data directly contrasting device-specific BEV designs remain limited. This represents an important gap in the literature, particularly as TAVR device selection is increasingly tailored to individual patient anatomy, procedural considerations, and anticipated performance tradeoffs. Moreover, available evidence remains largely confined to controlled trial settings, underscoring the need for complementary real-world data to better characterize BEV performance in routine clinical practice.

Accordingly, the present study aimed to provide a head-to-head comparison of procedural, in-hospital clinical, and echocardiographic outcomes following TAVR with the Edwards Sapien versus the MyVal BEVs using real-world data from a large, international registry.

## Methods

### Data Availability

The data that support the findings of this study will be shared on reasonable request to the corresponding authors.

### Study Design and Population

This was a retrospective observational study using data from the Aortic+Mitral TRAansCatheter Valve Registry (ClinicalTrials.gov Identifier: NCT04031274), an investigator-initiated, international, multicenter registry including over 23,000 TAVR procedures from 17 centers in Europe and Israel. The registry collects detailed baseline, procedural, and follow-up data. Local institutional review board approval was obtained at all participating centers, and data use complied with applicable ethical standards.

We included consecutive patients who underwent TAVR with a BEV-THV between registry initiation and August 31, 2025. Only procedures performed with contemporary BEV platforms were included: the Sapien 3, Sapien 3 Ultra, and MyVal THVs. Valve-in-valve procedures were excluded.

### Outcomes

The primary endpoint was all-cause 2-year mortality. Secondary endpoints included length of hospital stay and in-hospital adverse events defined by Valve Academic Research Consortium–3 criteria including permanent pacemaker implantation (PPI), stroke, major vascular and bleeding complications, and predischarge valve hemodynamics including mean/peak transvalvular gradient and paravalvular leak (PVL).

### Statistical Analysis

Continuous variables are reported as means (±SD) as well medians and interquartile ranges when appropriate. Categorical variables are described as n (%). Characteristics of study participants were compared using χ^2^ test for categorical variables and the Student’s t-test or Wilcoxon test, as appropriate for normal/nonnormal distributed continuous variables. All tests were 2-sided and a value of *p* < 0.05 was considered significant. Hazard ratios are accompanied by 95% CIs. Survival curves were plotted, and the Kaplan–Meier log rank test was used to test the study groups on survival at 2 years.

To control for variance in baseline characteristics, 2-stage propensity score matching was performed. A 1:3 cohort of MyVal and Sapien valves was constructed using nearest-neighbor propensity score matching with a caliper of 0.1. Matching quality was assessed by comparing standardized mean differences, with a standardized mean difference <0.2 indicating acceptable balance. The balance of covariates before and after matching was visualized using Love plots.

Cox proportional hazards models were used to assess the impact of the valve platform on survival after adjustment for covariates. Covariates were selected based on univariable analysis (*p* < 0.05). All analyses were performed using R (V.4.0.0; R-studio, Vienna, Austria).

## Results

In total, 1903 patients who underwent TAVR with BEV were included in the current study, comprising 1676 patients treated with the Edwards Sapien valve and 227 with the MyVal valve (Figure 1). Baseline characteristics of the entire cohort are summarized in Table 1. The mean age was 80.86 ± 6.9 years, with no significant difference between groups (Sapien 80.82 ± 6.93 vs. MyVal 81.15 ± 6.62; *p* = 0.498). The mean baseline transaortic gradients were comparable (44.89 ± 14.54 vs. 43.65 ± 16.55 mmHg; *p* = 0.245), as was surgical risk, with a mean Society of Thoracic Surgeons score of 3.87% ± 2.62% across both platforms (*p* = 0.647). Sex distribution, prevalence of small annulus, alternative access, prior percutaneous coronary intervention, bicuspid valve anatomy, left ventricular dysfunction, diabetes, hypertension, and atrial fibrillation were similar between groups. Patients treated with MyVal had lower rates of prior coronary artery bypass grafting (4.0% vs. 10.7%; *p* = 0.002) and baseline PPI (4.7% vs. 10.0%; *p* = 0.023). A numerical trend toward lower chronic obstructive pulmonary disease prevalence was observed in the MyVal cohort, although this did not reach statistical significance (8.4% vs. 13.1%; *p* = 0.057).

**Figure 1:**
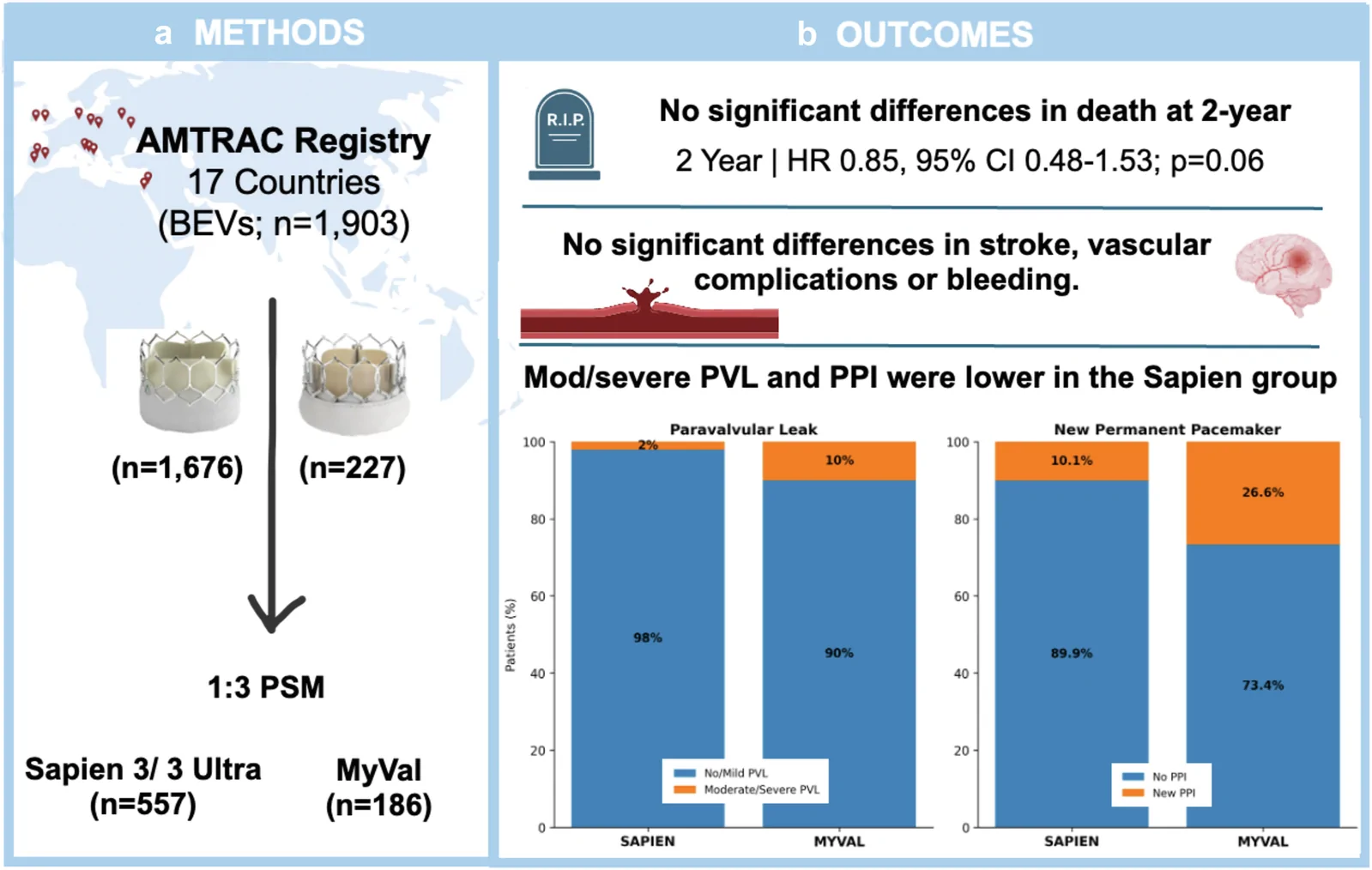
**Study design and outcomes.** Abbreviations: AMTRAC Registry, Aortic+Mitral TRAansCatheter Valve Registry; BEV, balloon-expandable valve; HR, hazard ratio; PPI, permanent pacemaker implantation; PSM, propensity score matching; PVL, paravalvular leak.

**Table 1:** Baseline characteristics of unadjusted cohort

	Overall (n = 1903)	Sapien (n = 1676)	MyVal (n = 227)	*p-*value
Age (mean [SD]), y	80.86 (6.90)	80.82 (6.93)	81.15 (6.62)	0.498
MG (mean [SD]), mmHg	44.74 (14.79)	44.89 (14.54)	43.65 (16.55)	0.245
STS (mean [SD])	3.87 (2.62)	3.86 (2.67)	3.95 (2.21)	0.647
Female (%)	670 (35.7)	595 (36.1)	75 (33.0)	0.414
Small aortic annulus (%)	650 (34.2)	579 (34.6)	71 (31.4)	0.385
Alternative access route (%)	59 (3.1)	54 (3.2)	5 (2.2)	0.530
Previous PCI (%)	453 (23.8)	407 (24.3)	46 (20.3)	0.211
BAV (%)	82 (4.3)	77 (4.6)	5 (2.2)	0.136
LV dysfunction (%)	351 (18.6)	302 (18.2)	49 (21.9)	0.214
COPD (%)	238 (12.5)	219 (13.1)	19 (8.4)	0.057
DM (%)	596 (31.4)	525 (31.4)	71 (31.4)	1.000
HTN (%)	1456 (78.1)	1299 (77.6)	157 (81.8)	0.224
AF (%)	415 (31.9)	355 (33.1)	60 (26.4)	0.062
Previous CABG (%)	188 (9.9)	179 (10.7)	9 (4.0)	0.002
Baseline pacemaker (%)	177 (9.5)	168 (10.0)	9 (4.7)	0.023

Abbreviations: AF, atrial fibrillation; BAV, bicuspid aortic valve; CABG, coronary artery bypass grafting; COPD, chronic obstructive pulmonary disease; DM, diabetes mellitus; HTN, hypertension; LV, left ventricular; MG, mean transvalvular gradient; PCI, percutaneous coronary intervention; STS, Society of Thoracic Surgeons predicted risk of mortality.

Notably, marked differences in THV size distribution were observed between platforms (Table 2). Among Sapien recipients, valve sizes were predominantly 23 mm and 26 mm, representing 31.32% (523) and 57.19% (955) of procedures, respectively. Smaller proportions received 20-mm (1.44%) or 29-mm (10.06%) valves. In contrast, MyVal demonstrated a broader size utilization, with intermediate diameters frequently used, including 21.5-mm (6.22%), 24.5-mm (24.44%), 27.5-mm (13.33%), and 30.5-mm (2.22%) valves. The most commonly implanted MyVal sizes were 24.5 mm, 23 mm, and 26 mm, with larger sizes such as 32 mm implanted in 8.0% of patients.

**Table 2:** THV size distribution of BEV

THV device size (mm)	Sapien (entire cohort; n = 1676)	MyVal (entire cohort; n = 227)	Sapien (matched cohort; n = 557)	MyVal (matched cohort; n = 186)
20	24 (1.44%)	3 (1.33%)	9 (1.62%)	3 (1.62%)
21.5	–	14 (6.22%)	–	11 (5.95%)
23	523 (31.32%)	46 (20.44%)	180 (32.37%)	40 (21.62%)
24.5	–	55 (24.44%)	–	44 (23.78%)
26	955 (57.19%)	39 (17.33%)	320 (57.55%)	33 (17.84%)
27.5	–	30 (13.33%)	–	23 (12.43%)
29	168 (10.06%)	15 (6.67%)	47 (8.45%)	14 (7.57%)
30.5	–	5 (2.22%)	–	4 (2.16%)
32	–	18 (8%)	–	13 (7.03%)

Abbreviations: BEV, balloon-expandable valve; THV, transcatheter heart valve.

Kaplan–Meier survival curves of up to 1 year for the propensity-matched cohort of 743 patients (Sapien n = 557; MyVal n = 186), are described in Figure 2. All-cause mortality at 1 year was 11% in the Sapien group and 9% in the MyVal group, whereas at 2 years, it was 19% and 15% in the Sapien and MyVal groups, respectively, with no significant difference between groups (*p* = 0.6). Procedural and in-hospital outcomes for the propensity-matched cohort are summarized in Table 3. In-hospital death occurred in 1.1% of patients, without significant variability between groups. Rates of vascular complications, bleeding events, acute kidney injury, stroke, cardiac tamponade, coronary obstruction, myocardial infarction, valve success, and in-hospital mortality were low and did not differ significantly between groups. New PPI occurred more frequently in MyVal recipients compared with Sapien (26.6% vs. 10.1%; *p* < 0.001) (Table 3). In addition, the length of hospital stay was longer in the MyVal group compared to the Sapien group (5.25 ± 5.62 vs. 3.79 ± 4.63 days; *p* < 0.001) (Table 3).

**Figure 2:**
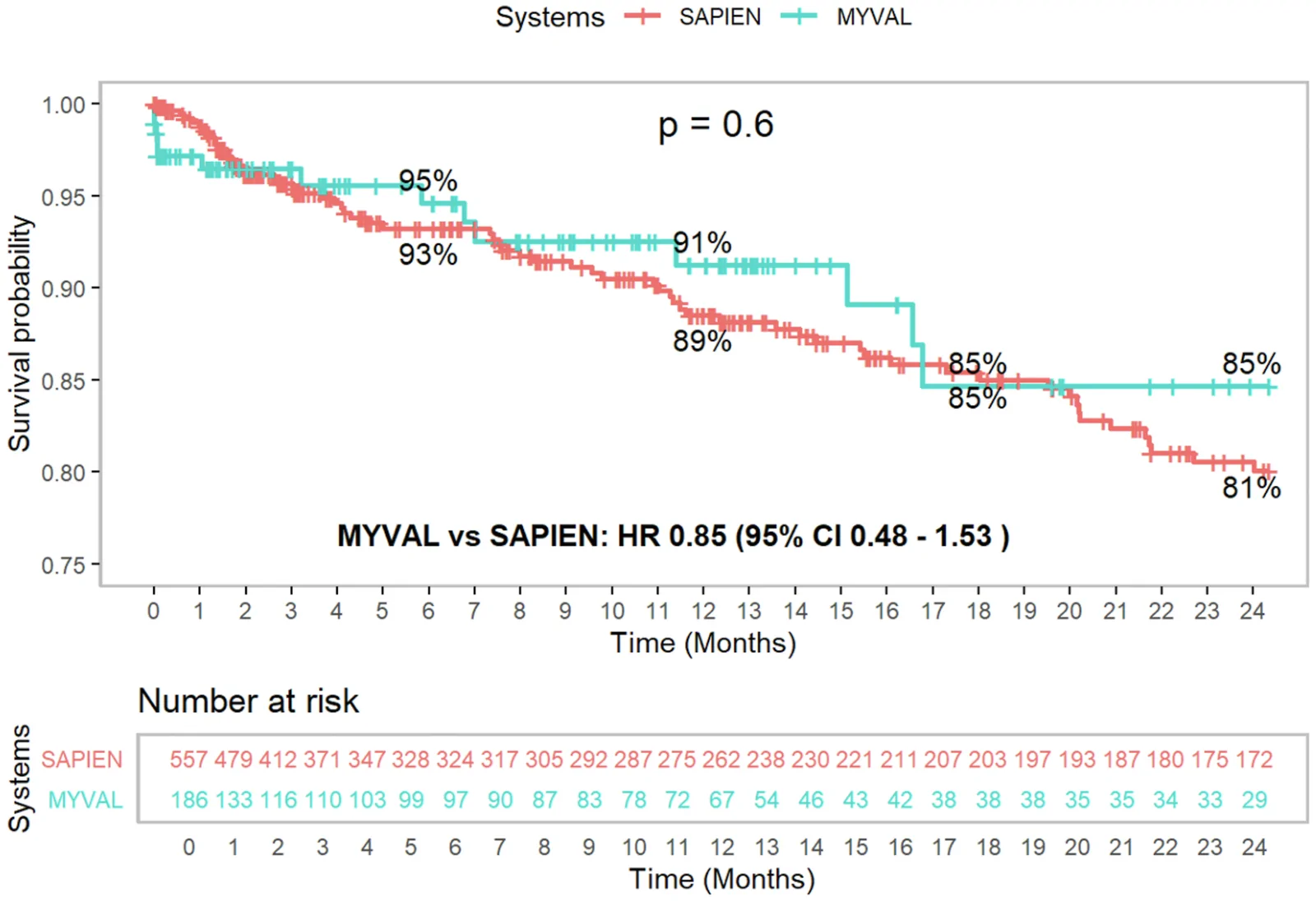
**Kaplan–Meier curves of 2-year survival after TAVR with different BEV platforms.** Abbreviations: BEV, balloon-expandable valve; HR, hazard ratio; TAVR, transcatheter aortic valve replacement.

**Table 3:** Outcomes of matched cohort

	Overall (n = 743)	Sapien (n = 557)	MyVal (n = 186)	*p-*value
Vascular complications				0.546
None (%)	651 (91.3)	483 (91.7)	168 (90.3)	
Minor (%)	43 (6.0)	29 (5.5)	14 (7.5)	
Major (%)	19 (2.7)	15 (2.8)	4 (2.2)	
Bleeding complications				0.222
None (%)	643 (90.3)	473 (89.9)	170 (91.4)	
Minor (%)	36 (5.1)	24 (4.6)	12 (6.5)	
Major (%)	23 (3.2)	20 (3.8)	3 (1.6)	
Life threatening (%)	10 (1.4)	9 (1.7)	1 (0.5)	
AKI (%)	40 (5.4)	33 (5.9)	7 (3.8)	0.346
Tamponade (%)	2 (0.4)	2 (0.6)	0 (0.0)	0.751
Coronary occlusion (%)	4 (0.6)	1 (0.2)	3 (1.6)	0.097
Valve success (%)	717 (96.6)	540 (96.9)	177 (95.7)	0.551
Stroke (%)	11 (1.5)	10 (1.8)	1 (0.5)	0.379
AMI (%)	2 (0.5)	0 (0.0)	2 (1.1)	0.474
PPI (%)	100 (14.3)	53 (10.1)	47 (26.6)	<0.001
In-hospital death (%)	8 (1.1)	5 (0.9)	3 (1.6)	0.683
LoS (mean [SD])	4.15 (4.93)	3.79 (4.63)	5.25 (5.62)	<0.001
MG (mean [SD])	10.22 (5.24)	10.45 (5.37)	9.84 (5.01)	0.234
PG (mean [SD])	18.92 (9.09)	19.75 (9.43)	17.55 (8.33)	0.014
PVL				<0.001
None (%)	305 (44.4)	232 (45.1)	73 (42.2)	
Mild (%)	354 (51.5)	271 (52.7)	83 (48.0)	
> mild (%)	28 (4.1)	11 (2.1)	17 (9.8)	
LV dysfunction (%)	138 (18.6)	103 (18.5)	35 (18.8)	1.000
MG > 10 mmHg (%)	178 (39.7)	125 (44.0)	53 (32.3)	0.019

Abbreviations: AKI, acute kidney injury; AMI, acute myocardial infarction; LoS, length of hospital stay; LV, left ventricle; MG, mean transvalvular gradient; PG, peak transvalvular gradient; PPI, permanent pacemaker implantation; PVL, paravalvular leak.

Postprocedural mean transvalvular gradients were similar between platforms (9.84 ± 5.01 mmHg for MyVal vs. 10.45 ± 5.37 mmHg for Sapien; *p* = 0.234) (Table 3, Figure 3A). Peak gradients were lower with MyVal (17.55 ± 8.33 vs. 19.75 ± 9.43 mmHg; *p* = 0.014) (Table 3, Figure 3B). Despite comparable mean gradients, a higher proportion of Sapien recipients exceeded the threshold for elevated postprocedural mean gradients (>10 mmHg) compared with MyVal (44.0% vs. 32.3%; *p* = 0.019) (Table 3). Distribution of PVL differed significantly between groups (*p* < 0.001), with more than mild PVL observed more frequently in the MyVal cohort (9.8% vs. 2.1%) (Table 3, Figure 3C). In Sapien recipients, no or mild PVL was achieved in 45.1% and 52.7%, respectively (Table 3). In MyVal recipients, no or mild PVL was achieved in 42.2% and 48%, respectively (Table 3).

**Figure 3:**
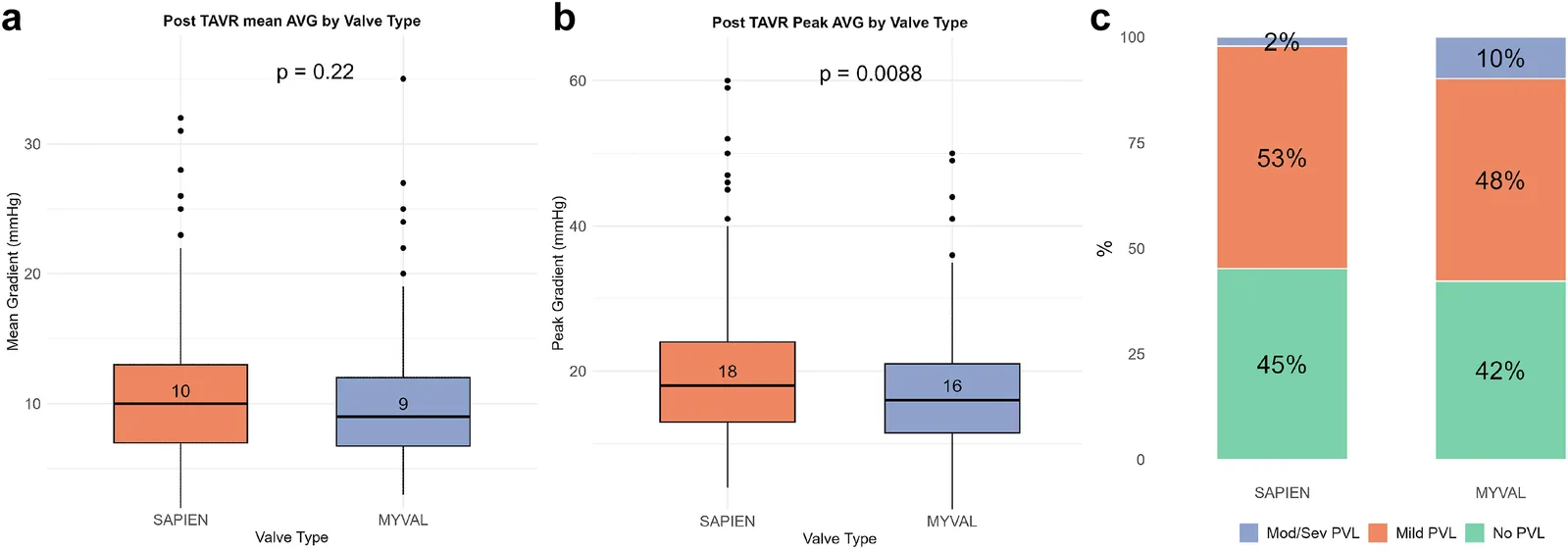
(a) The mean postprocedural transarotic gradient in the Sapien versus MyValve platforms; (b) peak postprocedural transarotic gradient in the Sapien versus MyValve platforms; (c) stacked bar plot representing the rate of paravalvular leak in the Sapien and MyVal groups. Abbreviations: AVG, aortic valve gradient; PVL, paravalvular leak; TAVR, transcatheter aortic valve replacement.

## Discussion

In this comparative analysis of balloon-expandable TAVR platforms from a multicenter international registry, several important differences between Sapien and MyVal emerged.

Valve size utilization differed substantially, with Sapien implants concentrated predominantly in 2 valve sizes, whereas MyVal was associated with a broader and more granular size distribution, including frequent use of intermediate and larger diameters. Although intermediate valve sizes offer the potential for more precise annular matching, real-world outcomes indicate that sizing alone may not be the primary determinant of procedural success. Additional studies are needed to refine patient selection and sizing strategies and better define the clinical value of intermediate sizing.

In the present study, despite these differences, short-term clinical outcomes were largely comparable. Two-year all-cause mortality did not differ significantly between groups, and rates of in-hospital death and major procedural complications were similar after propensity matching. However, MyVal implantation was associated with a significantly higher incidence of PPI and a longer length of hospital stay, suggesting potential differences in conduction system interaction or implantation depth.

Hemodynamic performance differed between platforms, with comparable postprocedural mean gradients but lower peak gradients observed with MyVal. Despite similar average mean gradients, a higher proportion of Sapien recipients exhibited mean gradients exceeding 10 mmHg, suggesting differences in gradient distribution. PVL also differed between the 2 THVs, with moderate or greater PVL occurring more frequently in the MyVal cohort.

Taken together, these data reinforce the concept that contemporary TAVR outcomes are influenced not only by valve class (BEV versus self-expandable valve), but also by device-specific design characteristics within each platform. Although randomized trials and large registries demonstrate broadly comparable survival and stroke outcomes between BEV and self-expandable valve systems, consistent differences in secondary endpoints highlight the importance of valve selection tailored to individual anatomical and clinical considerations.^6^^,^^7^

Historically, the BEV landscape has been dominated by a single reference platform, Sapien, and device selection within this class has often been considered largely interchangeable. The recent introduction of an additional THV system, MyVal, has expanded the armamentarium and challenged the notion of platform equivalence by incorporating distinct design features, including frame geometry, sealing mechanisms, expansion behavior, and size availability. MyVal, in particular, has attracted attention for its balloon-mounted valve design and the availability of intermediate sizes, providing greater flexibility to tailor valve selection to individual annular anatomies.

Beyond size options, the 2 platforms differ in frame composition and delivery system characteristics. MyVal is constructed from a nickel-cobalt alloy, whereas Sapien 3 uses cobalt-chromium. The MyVal valve is directly crimped onto the balloon and its delivery catheter lacks a pusher, which allows retrieval of the undeployed valve if necessary but may reduce device pushability, contributing to a higher rate of predilation compared with Sapien 3.^8^^,^^9^

The present study was therefore designed to investigate whether meaningful differences exist in procedural, clinical, and echocardiographic outcomes between contemporary BEV platforms using real-world data from a large international registry.

Our findings provide important context to the recently published COMPARE-TAVI 1 randomized trial,^5^ which represents the first head-to-head randomized comparison between MyVal/MyVal Octacor and the well-established Sapien 3 platform. Consistent with the COMPARE-TAVI 1 trial, our study demonstrates similar short-term clinical outcomes between Sapien and MyVal platforms, including in-hospital mortality and stroke. In COMPARE-TAVI 1, MyVal met noninferiority criteria compared with Sapien 3 for the composite endpoint of death, stroke, moderate or severe aortic regurgitation, or valve deterioration at 1 year. The concordance between randomized and real-world data reinforces the overall safety and effectiveness of both balloon-expandable platforms in contemporary TAVR practice.

A notable and consistent finding across studies is the higher rate of new PPI associated with the MyVal/Octacor/Octapro platform. In COMPARE-TAVI 1,^5^ first-time pacemaker implantation occurred in approximately 21% of MyVal-treated patients compared with 12% of the Sapien group, a pattern mirrored in our real-world cohort. The longer hospital stays observed in the MyVal group in our study may reflect the postprocedural conduction disturbances and higher pacemaker implantation rates. Valve frame design, radial force distribution, implantation depth, and operator familiarity with the new THV likely play contributory roles. These observations underscore the importance of careful implantation technique and continued refinement of device-specific procedural planning and execution strategies when using newer platforms.

Hemodynamic outcomes warrant nuanced interpretation. In our study, the mean postprocedural gradients were similar between Sapien and MyVal when assessed as continuous variables, consistent with the noninferiority design and findings of COMPARE-TAVI 1. However, a higher proportion of Sapien-treated patients exhibited elevated postprocedural mean gradients of over 10 mmHg. These findings align with COMPARE-TAVI 1, which reported smaller effective orifice area and higher mean gradients with Sapien 3 compared with MyVal. The MyVal design, including its leaflet configuration and availability of intermediate valve sizes, may facilitate more complete annular filling and improved leaflet opening in selected anatomies. Importantly, Sapien Ultra Resilia technology, associated with improved hemodynamics, was not included in the randomized trial or in our study, and its broader adoption may attenuate some of these observed differences.

In contrast to its favorable hemodynamic profile, MyVal was associated with a higher incidence of moderate or greater PVL, consistent with findings from the COMPARE-TAVI 1 trial^5^ and recent meta-analysis.^9^ This observation may reflect intrinsic differences in valve design, as the sealing skirt of the Sapien 3 valve may provide superior annular sealing, particularly in patients with irregular annular geometry or bulky and heavily calcified valve anatomy. In addition to design-related factors, operator experience and learning curve effects may have contributed to the higher PVL rates observed with MyVal, given its more recent introduction compared with the extensively adopted Sapien platform. Early experience with newer devices may influence implantation depth, sizing strategies, and postdilatation practices, all of which can affect sealing performance.

This study has several limitations. First, although propensity score matching and statistical adjustment mitigate selection bias, residual confounding from unmeasured variables remains an inherent limitation of the observational design, and cohort size reduction following matching may limit statistical power. Second, imaging assessments were not adjudicated by a centralized core laboratory, which may have resulted in interobserver variability in the evaluation of valve hemodynamics and PVL. Third, long-term outcomes beyond hospitalization were not available, precluding assessment of valve durability and late clinical events. Operator experience, implantation depth, and procedural nuances, factors known to influence conduction disturbances and hemodynamic performance, were not captured and may have affected outcomes. Fourth, the present study was not designed to specifically evaluate the impact of intermediate sizing of the MyVal/Ocatacor platform on clinical outcomes. Finally, the Sapien Ultra RESILIA valve was not included in this analysis. This latest-generation iteration incorporates modified leaflet attachment and RESILIA tissue technology, which have been associated with improved effective orifice area and potentially enhanced durability. As a result, the hemodynamic differences observed between Sapien and MyVal in this study may not fully reflect outcomes achievable with contemporary Sapien Ultra RESILIA technology, and our findings should be interpreted within this context.

## Conclusion

In this real-world comparison of BEV, Sapien, and MyVal demonstrated comparable early clinical outcomes. However, meaningful differences were observed in secondary endpoints, including higher rates of PPI and moderate or greater PVL with MyVal, alongside differences in gradient distribution and peak hemodynamics. These findings are consistent with observations from the COMPARE-TAVI 1 randomized trial. Notably, the absence of the Sapien Ultra RESILIA valve and MyVal Octacor/Octapro valve in this analysis limits extrapolation to the most contemporary platforms, which may offer improved hemodynamic performance and durability. Accordingly, valve selection in current practice should remain individualized, integrating patient-specific anatomy, conduction risk, sealing requirements, and lifetime management considerations, including future valve-in-valve feasibility. Further studies incorporating the latest-generation BEV technologies and longer-term follow-up are warranted, particularly in younger and lower-risk populations.

## Funding

The authors have no funding to report.

## Declaration of Generative AI and AI-Assisted Technologies in the Writing Process

Artificial intelligence tools, including ChatGPT (OpenAI), were used to assist in language editing, formatting, and improving the clarity of the manuscript. All content was reviewed and verified by the authors to ensure accuracy and integrity. No AI tools were used for data analysis, image generation, or interpretation of results.

## Disclosure Statement

Yeela Talmor-Barkan reports receiving speaker honoraria from Abbott. Amos Levi reports receiving speaker honoraria from Abbott and Boston Scientific. Marianna Adamo reports receiving speaker honoraria from Abbott and Edwards Lifesciences. Matti Adam reports receiving honoraria from Abbott, Boston Scientific, Edwards Lifesciences, JenaValve Technology, Medtronic, and Meril Life Sciences. Hendrik Wienemann reports consultancy fees and travel grants from JenaValve Technology. Rodrigo Estévez-Loureiro reports serving as consultant and proctor for Abbott Vascular, Edwards Lifesciences, Boston Scientific, Valgen, Jenscare, and Venus Medtech. Ignacio J. Amat-Santos reports receiving proctor and consultation fees from Boston Scientific, Medtronic, Meril Life, and MicroPort. Matjaz Bunc reports serving as proctor for Meril, Abbott, Edwards, and Medtronic.

The other authors had no conflicts to declare.

## References

[1] Cribier A., Litzler P.-Y., Eltchaninoff H. (2009). Technique of transcatheter aortic valve implantation with the Edwards-Sapien heart valve using the transfemoral approach. Herz.

[2] Mack M.J., Leon M.B., Thourani V.H. (2019). Transcatheter aortic-valve replacement with a balloon-expandable valve in low-risk patients. N Engl J Med.

[3] Abdel-Wahab M., Landt M., Neumann F.-J. (2020). 5-Year outcomes after TAVR with balloon-expandable versus self-expanding valves: results from the CHOICE randomized clinical trial. JACC Cardiovasc Interv.

[4] Duran Karaduman B., Ayhan H., Keleş T., Bozkurt E. (2021). Transcatheter tricuspid valve-in-valve implantation with a novel balloon expandable myval THV. Ann Thorac Surg.

[5] Terkelsen C.J., Freeman P., Dahl J.S. (2025). SAPIEN 3 versus Myval transcatheter heart valves for transcatheter aortic valve implantation (COMPARE-TAVI 1): a multicentre, randomised, non-inferiority trial. Lancet.

[6] Deharo P., Bisson A., Herbert J. (2020). Impact of Sapien 3 balloon-expandable versus evolut R self-expandable transcatheter aortic valve implantation in patients with aortic stenosis: data from a nationwide analysis. Circulation.

[7] Lerman T.T., Levi A., Kornowski R. (2023). Meta-analysis of short- and long-term clinical outcomes of the self-expanding Evolut R/pro valve versus the balloon-expandable Sapien 3 valve for transcatheter aortic valve implantation. Int J Cardiol.

[8] Seth A., Kumar V., Singh V.P., Kumar D., Varma P., Rastogi V. (2023). Myval: a novel transcatheter heart valve for the treatment of severe aortic stenosis. Interv Cardiol.

[9] Bhasin D., Bhasin A., Sangdup T., Sharma A., Panda P., Paul Sharma Y. (2025). Early outcomes of transcatheter aortic valve implantation for severe aortic stenosis with Myval versus Edwards Sapien 3 valve: a systematic review and meta-analysis. Cureus.

