# Supplementary material for: Real-World Comparison of Balloon-Expandable Valves for Transcatheter Aortic Valve Replacement: Data From the Aortic+Mitral TRAansCatheter Valve Registry
Source: Struct Heart. 2026 Jul 27;10(10):101104. doi: 10.1016/j.shj.2026.101104 (PMC13628656; doi:10.1016/j.shj.2026.101104)
Supplement: Supplementary file 1 [file mmc1.docx]

**eTable 1. Baseline characteristics of matched cohort**

|  | **Overall**  **(n = 743)** | **Sapien**  **(n = 557)** | **MyVal**  **(n = 186)** | **p-value** |
| --- | --- | --- | --- | --- |
| Age (mean [SD]), years | 80.64 (6.68) | 80.59 (7.04) | 80.79 (5.48) | 0.722 |
| MG (mean [SD]), mmHg | 44.18 (15.73) | 44.24 (15.32) | 43.98 (16.95) | 0.843 |
| STS (mean [SD]) | 3.93 (2.68) | 3.93 (2.84) | 3.90 (2.14) | 0.877 |
| Female (%) | 261 (35.1) | 198 (35.5) | 63 (33.9) | 0.744 |
| Small aortic annulus (%) | 257 (34.6) | 195 (35.0) | 62 (33.3) | 0.744 |
| Alternative access route (%) | 7 (0.9) | 5 (0.9) | 2 (1.1) | 1.000 |
| Previous PCI (%) | 186 (25.0) | 142 (25.5) | 44 (23.7) | 0.687 |
| BAV (%) | 21 (2.8) | 16 (2.9) | 5 (2.7) | 1.000 |
| LV dysfunction (%) | 138 (18.6) | 103 (18.5) | 35 (18.8) | 1.000 |
| COPD (%) | 59 (7.9) | 44 (7.9) | 15 (8.1) | 1.000 |
| DM (%) | 234 (31.6) | 173 (31.2) | 61 (33.0) | 0.715 |
| HTN (%) | 599 (80.6) | 446 (80.1) | 153 (82.3) | 0.585 |
| AF (%) | 197 (33.2) | 142 (34.8) | 55 (29.6) | 0.245 |
| Previous CABG (%) | 72 (9.7) | 65 (11.7) | 7 (3.8) | 0.003 |
| Baseline pacemaker (%) | 53 (7.1) | 44 (7.9) | 9 (4.8) | 0.213 |

Abbreviations: AF, atrial fibrillation; BAV, bicuspid aortic valve; CABG, prior coronary artery bypass grafting; COPD, chronic obstructive pulmonary disease; DM, diabetes mellitus, HTN, hypertension; LV, left ventricular; MG, mean transvalvular gradient; PCI, percutaneous coronary intervention; STS, Society of Thoracic Surgeons predicted risk of mortality.

**eTable 2. Outcomes of unadjusted cohort.**

|  | **Overall**  **(n = 1903)** | **Sapien**  **(n = 1676)** | **MyVal**  **(n = 227)** | **p-value** |
| --- | --- | --- | --- | --- |
| Vascular complications |  |  |  | 0.237 |
| None (%) | 1652 (90.2) | 1446 (89.9) | 206 (92.0) |  |
| Minor (%) | 110 (6.0) | 96 (6.0) | 14 (6.2) |  |
| Major (%) | 70 (3.8) | 66 (4.1) | 4 (1.8) |  |
| Bleeding complications |  |  |  | 0.125 |
| None (%) | 1640 (89.5) | 1430 (89.0) | 210 (92.5) |  |
| Minor (%) | 90 (4.9) | 78 (4.9) | 12 (5.3) |  |
| Major (%) | 66 (3.6) | 63 (3.9) | 3 (1.3) |  |
| Life threatening (%) | 37 (2.0) | 35 (2.2) | 2 (0.9) |  |
| AKI (%) | 109 (5.7) | 98 (5.8) | 11 (4.8) | 0.648 |
| Coronary occlusion (%) | 7 (0.4) | 4 (0.2) | 3 (1.3) | 0.060 |
| Valve success (%) | 1819 (95.7) | 1601 (95.6) | 218 (96.5) | 0.691 |
| Stroke (%) | 41 (2.2) | 37 (2.2) | 4 (1.8) | 0.849 |
| AMI (%) | 2 (0.3) | 0 (0.0) | 2 (1.1) | 0.127 |
| PPI (%) | 229 (13.2) | 180 (11.6) | 49 (26.8) | <0.001 |
| In-hospital death (%) | 37 (1.9) | 33 (2.0) | 4 (1.8) | 1.000 |
| LoS (mean [SD]) | 3.95 (18.04) | 3.87 (19.11) | 4.54 (5.57) | 0.604 |
| MG (mean [SD]) | 10.49 (4.89) | 10.70 (4.84) | 9.53 (5.00) | 0.002 |
| PG (mean [SD]) | 19.76 (8.55) | 20.22 (8.52) | 17.42 (8.35) | <0.001 |
| PVL (%) |  |  |  | <0.001 |
| None | 841 (48.3) | 752 (49.2) | 89 (41.6) |  |
| Mild | 843 (48.4) | 738 (48.3) | 105 (49.1) |  |
| > mild | 57 (3.3) | 37 (2.5) | 20 (9.3) |  |
| LV dysfunction (%) | 351 (18.6) | 302 (18.2) | 49 (21.9) | 0.214 |
| MG >10mmHg (%) | 486 (43.3) | 424 (46.2) | 62 (30.2) | <0.001 |

Abbreviations: AKI, acute kidney injury; AMI, acute myocardial infarction; LoS, length of hospital stay; LV, left ventricle; MG, mean transvalvular gradient, PG, peak transvalvular gradient; PPI, permanent pacemaker implantation; LoS, length of hospital stay; PVL, paravalvular leak.
